# At war or saving lives? On the securitizing semantic repertoires of Covid-19

**DOI:** 10.1177/00471178221122957

**Published:** 2022-09-23

**Authors:** Stephane J Baele, Elise Rousseau

**Affiliations:** University of Exeter; University of Namur

**Keywords:** Covid-19, language, methods, securitization, semantic repertoire, speeches

## Abstract

This paper offers a multi-dimensional analysis of the ways and extent to which the US president and UK prime minister have securitized the Covid-19 pandemic in their public speeches. This assessment rests on, and illustrates the merits of, both an overdue theoretical consolidation of Securitization Theory’s (ST) conceptualization of securitizing language, and a new methodological blueprint for the study of ‘securitizing semantic repertoire’. Comparing and contrasting the two leaders’ respective securitizing semantic repertoires adopted in the early months of the coronavirus outbreak shows that securitizing language, while very limited, has been more intense in the UK, whose repertoire was structured by a biopolitical imperative to ‘save lives’ in contrast to the US repertoire centred on the ‘war’ metaphor.

## Introduction

In October 2019, exactly 45 days before the first Covid-19 case was detected, the Nuclear Threat Initiative and the Johns Hopkins Center for Health Security launched a brand-new benchmarking effort aiming at assessing health security across the 195-state parties to the International Health Regulations. The Global Health Security Index ranked states according to their level of preparedness to deal with serious outbreaks. In this list, the United States of America ranked first, the United Kingdom second. Yet, by summer 2020, both countries ranked in the top-10 of the states the worst hit by the Covid-19 pandemic. Much of the controversy that ensued centred on whether the two governments had initially downplayed the risk or/and been inconsistent in their decisions – and, crucially, communication – about the pandemic.

The present article sheds light on this puzzle, from the specific angle of Securitization Theory (ST). More precisely, we offer a multidimensional evaluation of the *intensity* and *way* in which the US President and UK Prime Minister have *securitized* the Covid-19 pandemic in their public speeches. In other words, how they framed it as an urgent security threat requiring extraordinary measures. This assessment rests on, and illustrates the merits of, an overdue consolidation of ST’s conceptualization of securitizing language, which allows us to compare and contrast the two leaders’ respective ‘securitizing semantic repertoires’ – the specific combination of words they adopted in the first months of the coronavirus outbreak to depict the virus as a security threat – and measure the intensity of their securitizing language over time.

In doing so, this research uncovers two main findings. First, the *intensity* of securitizing language in both countries was surprisingly low. We show that while both the US President and UK Prime Minister did securitize the Covid-19 pandemic in their public speeches, they did not make an extensive use of securitizing language – with the exception of some noticeable and widely mediatized spikes. Second, we reveal a paradox: while the intensity of the securitizing language has consistently been higher in the UK, it is in the US that the discourse of hard security has been more prominent. To investigate this, we undertake a granular analysis of each securitizing semantic repertoire and show that this variation is explained by a difference in the *way* each leader securitizes the issue. In particular, we show a variation in the referent object of Johnson and Trump, that is, what is seen as ‘existentially threatened’ and as having ‘a legitimate claim to survival’.^1^ Indeed, while the UK’s securitizing repertoire has been systematically structured by the biopolitical imperative of ‘saving lives’, the US’ repertoire is characterized by the use of the war metaphor. Perhaps counterintuitively, therefore, we show that using the war metaphor does not necessarily mean that the overall discourse over an issue will be highly securitized.

These findings might come as a surprise since the pandemic seems to present a textbook case of securitization, with extraordinary measures being implemented after state leaders pronounced powerful speech acts presenting the disease as a fundamental threat. As the canonical formulation indeed goes, securitization happens when ‘an issue is presented as an existential threat, requiring emergency measures and justifying actions outside the normal bounds of political procedure’.^2^ On the one hand, as the virus accelerated its propagation in the months of January–May 2020, almost every single government across the globe took extraordinary emergency measures of a kind and scope unseen during 20th century peacetime: drastic lockdowns were ordered, massive liquidity was injected in national economies, the army was deployed in the streets, borders were closed, etc. On the other hand, the governments who deployed these measures were keen to present the disease as a security threat to be tackled urgently. To take a few examples, on 16 March 2020, Emmanuel Macron proclaimed France to be ‘at war’, stressing the unprecedented nature of his ‘decisions in time of peace’ and arguing that ‘all these measures are necessary for our security’.^3^ Benjamin Netanyahu announced Israel to be at ‘war with an invisible enemy, the virus’, and Abdullah II claimed – in military uniform – that each Jordanian ‘is a soldier’ in the battle against the epidemic.^4^ The Covid-19 case thus appears to closely correspond to the ideal-type of securitization.

With its seemingly clear-cut sequence of securitization,^5^ the Covid-19 case is an opportune starting point for strengthening ST’s account of what is arguably its pivotal tenet: the question of what exactly constitutes ‘securitizing’ language. Surprisingly indeed, the substance of what Buzan, Waever and De Wilde initially called the ‘rhetoric of existential threat’,^6^ that is, the language used by a securitizing actor to move an issue from ‘normal’ politics to the realm of exceptional security politics, has been largely underspecified in securitization studies.^7^ Building on existing, but partial, attempts to describe the semantic dimension of this language, we suggest a consolidated framework for the study of ‘securitizing semantic repertoires’, which comes with a tailored methodology. This approach clarifies what securitizing language is and explains how to systematically study it, paving the way for a finer understanding of the many instances where political leaders justify extraordinary policies with the rhetoric of security threat (in realms as diverse as immigration, religious freedom or climate change). The granular unpacking of the various formal dimensions of securitizing language clears thus the path for a finer understanding of why this type of language is such a frequent and powerful type of communication.

This endeavour proceeds in three steps, which correspond to the three contributions of the paper (theoretical, methodological and empirical). First, we explain why more precision is needed in ST’s description of securitizing language, especially its lexical and semantic dimensions, and we consolidate the already-existing concept of securitizing ‘semantic repertoire’ to remedy this problem. Second, we explain the mixed-method approach used to analyse these repertoires. The approach consists in the use of two computer-assisted tools for content analysis (a co-occurrence network and a dictionary-based measurement), complemented with qualitative readings of significant texts. This combination strengthens and enriches the panel of methods usually employed to study securitization, and offers a solution to the underdevelopment of methods in securitization research.^8^ We detail and justify how we applied our methodology, which enables both diachronic and synchronic analyses, to the two cases evoked above: the public speeches on Covid-19 of Donald Trump, and Boris Johnson.^9^ Third, we present our results and discuss our findings, offering both important insights on the Covid-19 case, and empirical evidence for our theoretical intervention. By laying bare the semantic markers of what appears to be the most remarkable instantiation of securitization in recent times, we also enrich the literature on the securitization of global health.^10^

## Theoretical framework: what ‘speaking security’ really is

Language plays a central role in the securitization process conceptualized by the Copenhagen School.^11^ Speech acts are indeed the means used by a securitizing actor to convince an audience of the ‘critical vulnerability of a referent object’, that is, for example, the state, society, an institution or another referent group, ‘by investing the referent subject with such an aura of unprecedented threatening complexion that a customized policy must be undertaken immediately to block its development’.^12^ The pivotal moment in this securitizing move sequence is the securitizing speech act. As Williams clearly describes, ‘issues become “securitized”, treated as security issues, through these speech acts which do not simply describe an existing security situation, but bring it into being as a security situation by successfully representing it as such’.^13^ The emphasis on one-shot speech acts ‘expresses a more recognizable political investment’^14^ than other ST variants investigating the impact of language games, broader narratives or discursive environments on security policies.^15^ But these other approaches still attribute to words the power of shaping threat perceptions and security preferences.

Yet, in spite of the centrality of language and ST scholars’ in-depth study of the illocutionary and perlocutionary foundations of securitizing speech acts, what this language actually looks like, lexically, remains unspecified. In other words, the *semantic* dimension of securitizing language, the way words convey the securitizing meaning, has not been conceptualized precisely. In the initial formulation of ST, Waever claimed that ‘the word “security” is the act’.^16^ In their seminal book, Buzan, Waever and De Wilde added that securitization necessitated ‘a rhetoric of existential threat’^17^ that is not necessarily ‘defined by uttering the word security’; there are ‘instances in which the word security appears without this logic and other cases that operate according to that logic with only a metaphorical security reference’.^18^ Although such an under-specification of securitizing language may well have been intentional, probably reflecting the acknowledgement that it varies across contexts, a more in-depth and formal exploration of the potentially recurring features of this rhetoric, what it can look like, what its major lexical markers can be, was warranted. Surprisingly, such an exploration was yet to be done in a systematic way, contrasting with the kind of work done in other fields of critical security studies (e.g. constructivist feminism).

Our aim here is to offer a more detailed, formal and empirically operational conceptualization of securitizing language. While quite a few scholars have offered depictions of this language in action,^19^ three substantial contributions have paved the way for our framework. First, Balzacq acknowledged in his 2005 article that securitizing language was multifaceted, chiefly because a securitizing actor can use a series of different ‘heuristic artifacts’ (analogies, metaphors, metonymies and stereotypes) to gain traction.^20^ Together, these particular formulas constitute a ‘semantic repertoire of security’ that contains a cultural dimension.^21^ In his 2011 volume, Balzacq further highlighted metaphors and underlined the importance of the ‘semantic regularity’ of ‘repertoires of security’.^22^ In the same volume, Vultee’s assimilation of securitizing language to media frames paved the way for a more detailed lexical analysis of the words used, more or less strategically, by securitizing actors when constructing an issue as a threat.^23^ Second, Klüfers adopted a socio-pragmatist perspective to further define Balzacq’s ‘repertoires’. Defining acts of securitization as ‘discursive processes which evolve through one or more “security repertoires” [. . .which. . .] are systematically related sets of terms’. He stressed that, instead of a single ‘security repertoire’ being common to all cases of securitization, each instance of securitizing language rests on a particular combination of words ‘organized around one or more central metaphors’.^24^ In line with Klüfers, securitization scholars thus ought to provide fine-grained analyses of these specific sets of words that together construct the meaning of threat attached to the referent object. Third, Baele and Sterck argued that there is no such thing as a ‘pure’ securitizing language but rather ‘framing narratives whose securitising intensity may be more or less strong’.^25^ Noting that each speech may contain a smaller or greater number of words that belong to the semantic field of security (e.g. ‘threat’, ‘security’, ‘fight’, ‘war’), they suggested measuring the saliency of this vocabulary to evaluate the intensity of the linguistic securitizing move. To do so, as explained below, they created a ‘Security Lexicon’ (SL) of 222 words unambiguously pertaining to the ‘hard’ security lexical field.

We consolidate the findings of these contributions into a coherent theoretical framework for the study of securitizing semantic repertoires. Our framework concentrates on one specific step in the securitization process, leaving aside what comes before (decisions to securitize, path-dependencies leading to the speech act, etc.) and after (impact of the language on the audience, adoption of extraordinary measures, possible contestations of the securitization, etc.). We do not aim to offer an overview of the whole process, but rather to clarify as much as possible its pivotal moment.^26^ This framework articulates what we suggest are the five main formal semantic dimensions of securitizing repertoires, based on the above literature as well as influential scholarship in the various fields studying language in social action: (1) ‘generic’ versus ‘contingent’ lexicons, (2) stylistic devices, (3) parts of speech, (4) associative networks and (5) semantic context. Table 1 above lists these dimensions, which are unpacked in the following paragraphs.

**Table 1.** The five dimensions of securitizing semantic repertoires.

*Generic* Lexicon and *Contingent* Lexicon
Stylistic devices
Parts of speech
Associative network
Semantic context

### Generic and contingent lexicons

We argue that a securitizing semantic repertoire is neither fully culturally dependent, nor totally universal. Each repertoire contains, on the one hand, some of the ‘hard security’ words making Baele and Sterck’s SL (e.g. ‘war’, ‘threat’, ‘security’). These *generic* words are those that directly evoke a security threat regardless of the context. On the other hand, securitizing semantic repertoires also contain *contingent*, less directly and obviously securitizing terms that can nonetheless have a securitizing effect because of their particular socio-cultural resonance. For example, the words ‘powerful chemical agent’ might not be immediately evident, generic securitizing words, but may nonetheless be highly securitizing in a place such as Salisbury, UK, where Sergei Skripal was poisoned with a nerve agent in 2018. Even generic security words are not universally impactful: their effect on the audience can vary across socio-cultural settings. In sum, we suggest that any given securitizing semantic repertoire is a particular combination of what we call a ‘generic lexicon’ (which corresponds to SL) and a ‘contingent lexicon’ (which is particular to each case), each time of varying proportions. Attuning, for each securitizing speech, to the saliency of generic lexicon, and simultaneously identifying which words constitute the contingent lexicon, provides rich information into the working mechanisms of security persuasion.

### Stylistic devices

Securitizing repertoires typically contain, and are usually organized around, stylistic devices like metaphors, historical analogies or personification. Each plays a particular role in the rhetorical process, and serves as anchor to either the contingent or generic repertoires. First, research in linguistics and social cognition consistently shows how important *metaphors* are in the construction of social and political meaning^27^ – and this holds true in securitization. As Lakoff summarizes, metaphors are mental shortcuts, allowing to quickly ‘conceptualize one mental domain in terms of another’.^28^ In this process, a particular meaning, coming from the root mental domain, is attached to a new domain, in such a way that any moral evaluations and normative preferences pertaining to the root domain is assigned to the new one. Referring to migration as a wave, for example, or terrorism as a cancer, offers simplified understandings of these complex phenomena which imply specific solutions and trigger particular emotions among the audience. The war metaphor, in particular, has already been identified as a major rhetoric device to securitize issues, such as drugs or crime.^29^ This is because war metaphors ‘draw on basic and widely shared schematic knowledge that efficiently structures our ability to reason and communicate about many different types of situations, and reliably express an urgent, negatively valenced emotional tone that captures attention and motivates action’.^30^ Not only do war metaphors evoke a sense of fear which ‘can motivate people to pay attention, change their beliefs, and take action about important social issues’,^31^ they also suggest that the efficient reaction to the issue against which a ‘war’ is waged is necessarily uncompromising. War metaphors refers to a clear hierarchy of command, action and obedience. They also signal urgency, and the risk of a weak reaction.^32^ As such, ‘war’ is not only an unambiguous security word of the generic lexicon, it is also a convenient, and thus prominent, securitizing metaphor.

Second, besides metaphors, with which they share the same comparative structure, *historical analogies* are a recurring component of political decision-making and discourse,^33^ which can play a significant role in securitizing semantic repertoires. They confer meaning to a new situation by transposing a simplified interpretation of a past event onto a current one, and are thus important in two ways when it comes to securitization. Firstly, by invoking collective memory, they have the potential to bring together the community as an in-group being threatened by the referent object. Secondly, analogies serve not only a diagnostic function (explaining what is happening), but also a prescriptive and moral function (suggesting what needs to be done, and what is good to do given the ‘lessons’ from the past).^34^ For example, by claiming that a given issue creates ‘a new Verdun’, a French official is immediately understood by his/her audience as portraying that issue as an attack of extreme violence that necessitates all forces of the nation to be redirected towards that single focal point. A British leader referring to the Blitz would suggest similar meaning and solutions. These two examples incidentally show the importance of the contingent lexicon in securitization: culturally resonant words not belonging to the generic security lexicon (e.g. ‘Verdun’) can be used to amplify or dramatize securitizing speech acts.

Third, *personification* is another recurring stylistic device in political rhetoric,^35^ notably in its visual expressions, that can be used to enhance a securitizing move in two different ways. One possibility is to assign human characteristics and agency to an abstract and non-agential threat, which makes it more tangible to the audience. The other possibility is to personify the group threatened by the issue, which can reinforce a sense of common, threatened identity among the audience, as well as conveniently locate the blame into a stereotypical ‘other’. Nazi propagandists were, for instance, very prolix users of personification, which at times morphed into animalization when it came to their enemies.

We suggest that stylistic devices such as metaphors, historical analogies and personification cannot be ignored by securitization scholars who wish to understand why particular securitizing speech acts are used or gain traction.

### Parts of speech

Words play a role in securitizing language without necessarily setting up a stylistic device or evoking security. Indeed, attuning to the specific roles of the different ‘parts of speech’ (nouns, verbs, adjectives, adverbs, pronouns, prepositions, conjunctions, interjections and articles) is crucial in understanding the semantic dimension of securitizing repertoires, as indeed any political statement.^36^*Adverbs* and *adjectives* do not belong to the generic security lexicon, yet they can decisively strengthen securitizing semantic repertoires by reinforcing the sense of urgency and threat, and by enhancing the impression that extraordinary measures need to be taken. A speech that makes a repeated use of adverbs like ‘absolutely’, ‘uncompromisingly’ or ‘urgently’, and adjectives like ‘life-threatening’, ‘dangerous’ or ‘unprecedented’, conveys a more powerfully securitizing meaning than one that does not. Adjectives help depict and specify the nature of the situation by characterizing protagonists and actions in ways that can construct very specific threatening pictures. For example, characterizing the securitizing subject as ‘vicious’ will entail different perceptions among the audience than depicting it as ‘brutal’. *Verbs* that denote a swift action, such as ‘react’, ‘counter-attack’ or ‘move’, reinforce the urge to securitize more than non-action verbs such as ‘ponder’, ‘consider’ or ‘collaborate’; their use is therefore more likely in securitizing semantic repertoires. Finally, *pronouns* also are of utmost importance in conveying emotions and sense of belonging in speech.^37^ The use of ‘we’ by political leaders is particularly significant as it performatively creates a sense of common in-group identity. In contrast, employing ‘they’ can conglomerate non-members into a single out-group.

In sum, attuning to the frequency and positioning of parts of speech is key, not only to evaluating the power of securitizing repertoires, but also to identifying the particular securitizing objects and subjects they construct.

### Associative networks

The presence of stylistic devices and parts of speech is not enough to convey a securitizing message. Rather, it is the relationship between these units, how they are organized together to convey meaning, that truly constitutes securitizing semantic repertoires. As Kunda and other cognitive scientists emphasize, the meaning of a message results from its ‘interconcept organization’, in other words, from the ‘associative network’ whereby concepts composing the message take their meaning from the other concepts that accompany them.^38^ The regular co-occurrence of a set of words in a series of messages primes the audience to understand these concepts as linked, and provides the basic structure of the meaning of a situation or issue. For example, the systematic co-occurrence of the word ‘mosque’ with concepts like ‘attack’, ‘terrorist’ and ‘war’ in white supremacist blogs discussing the Ground Zero mosque initiative built an understanding of the project as a fundamental security threat, an interpretation that permeated mainstream media and public opinion.^39^ Similarly, the relentless association by Salafi-jihadist propagandists of the out-group labels ‘Crusader’ and ‘Americans’ with descriptions of children killed by airstrikes creates an image of an exceptionally cruel enemy consistently harming the most vulnerable of their in-group. Securitization scholars thus ought to attune not only to lexical units of analysis but also to how they are connected together to create the particular meaning of an issue through priming – and thus imply a particular desired response.^40^ In that way, important insights can be gained regarding the particular ways securitizing speech acts construct the threat: what is/are the threatened objects, what constitutes the broader logic and domain of the threat (a territorial logic, a cultural or identity logic, a biological logic, etc.), and much more.

### Semantic context

Finally, securitizing semantic repertoires rarely occur in isolation from other repertoires. Securitizing repertoires generally co-exist within a given speech/text with non-securitizing language, which can have implications for how the message is understood and interpreted by the audience: indeed diluting a securitizing repertoire within a long intervention blending other repertoires could carry less weight than entirely containing the allocution within the boundaries of the securitizing repertoire.^41^ This is because, as soon as another repertoire is used, the overall meaning of the speech starts to be determined by a wider associative network of concepts. In other words, what matters is not just if, but how much, proportionally, ‘security words’ are uttered, and how they build, with coexisting repertoires, a more or less threatening picture of a situation.

**Figure 1.**
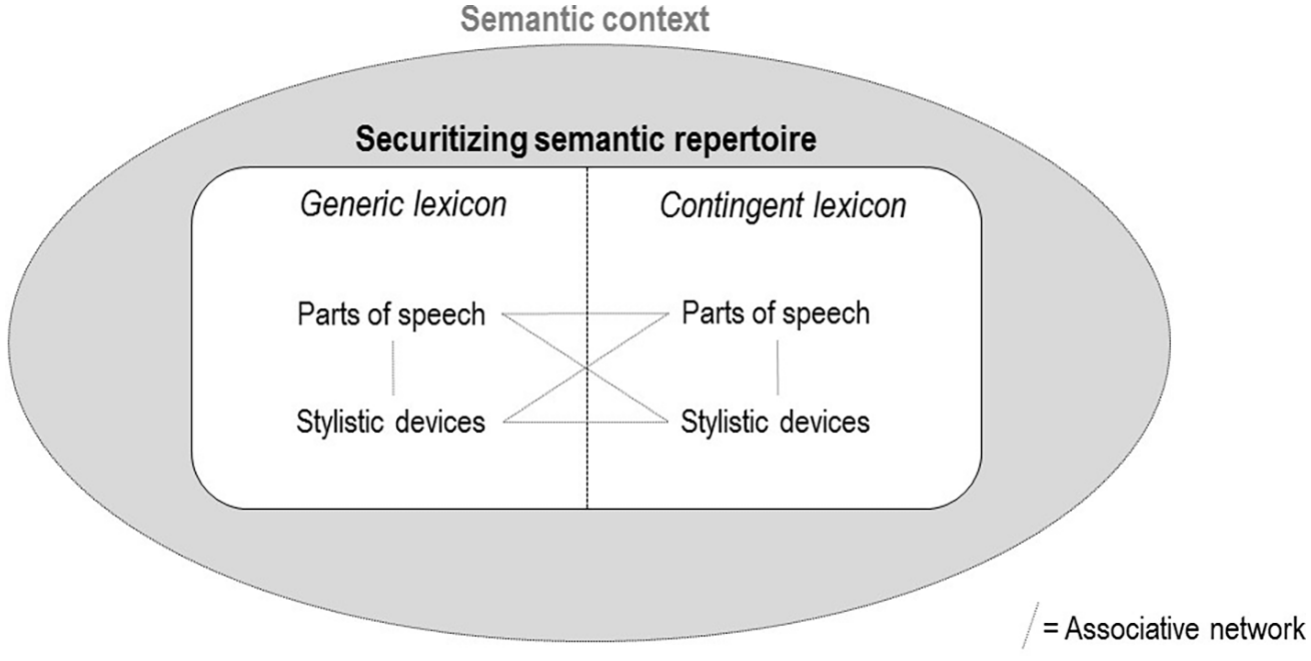
A schematic representation of a securitizing semantic repertoire.

The five dimensions are schematically represented together in Figure 1 below: securitizing semantic repertoires are situated within a broader semantic context, and contain, to varying extents, generic and contingent lexicons, each of which made of a particular combination of parts of speech and stylistic devices interlinked within an associative network. Before applying this multi-dimensional model of securitizing security repertoires to the Covid-19 case to demonstrate its empirical utility, we explain in the next section how each element can be analysed.

## A mixed-method analysis of securitizing semantic repertoires

To study securitizing semantic repertoires, we suggest a methodology based on the combination of two existing computational tools for the study of textual content – dictionary-based approaches and co-occurrence networks – together with a close qualitative reading of particular interventions identified as important by the quantitative inquiry. Dictionary-based approaches and co-occurrence networks are closely related (they both compute word frequencies) but each captures different dimensions of semantic repertoires. They therefore answer different questions and should be combined to offer a granular analysis.

This mixed-methods approach embraces the practice of ‘triangulation’ in security studies.^42^ In particular, it rests on the conviction that quantitative content analysis can powerfully complement the qualitative techniques usually employed by securitization scholars. More specifically, it follows calls to ‘diversify [ST’s] methodological toolbox’ towards quantitative methods.^43^ The present effort thus stands in line with previous contributions that successfully combined computational content analysis and critical discourse analysis,^44^ diversifying the existing effort to fruitfully deploy multi-method designs in securitization research (such as those combining discourse analysis with process-tracing or public opinion surveys)^45^ in order to address securitization research’s ‘bias in favour of high-level theorizing and evaluative procedures at the detriment of empirics-driven knowledge relying on constructive procedures’.^46^

### First step: dictionary-based approach

The first step is to use a dictionary measuring the intensity of the *generic lexicon* of the securitizing semantic repertoire. Dictionary-based approaches are commonly used to calculate the weight of a given list of theoretically significant words in a corpus. Dictionaries have been built to measure the emotional tone of authoritarian leaders’ speeches,^47^ the saliency of racial slurs in texts,^48^ or variations in terrorist ideologies over time,^49^ among others. As noted above, a dictionary evaluating the intensity of securitizing language already exists: Baele and Sterck’s Security Lexicon (SL) has been developed to ‘measure the saliency within texts of the security “semantic regularity”’, whereby ‘the more a political actor makes use of words taken out of this set, the more his or her narrative establishes a securitising move’.^50^

We use the Linguistic Inquiry and Word Count (LIWC)^51^ to calculate the ratio of the words making the SL out of the total number of words for each one of the 126 segments constituting our corpus (see below).^52^ This allows us to compare variations of this ratio in each one of these segments. In doing so, we follow Smith, Stohl and al-Gharbi’s demonstration that the SL is best used diachronically to reveal shifts in the intensity of securitizing language over time.^53^ Measuring the saliency of generic security words in such a way importantly approximates the strength of the securitizing speech act (does the speaker merely mentions a threat once, or does s/he relentlessly repeat it?) and provides a clear indication as to whether or not securitization is a sporadic linguistic practice or a sustained one.

This first step thus serves three goals: to gain a general overview of the intensity of the generic lexicon in our securitizing semantic repertoires, to trace the evolution of this lexicon across time, and to identify texts warranting further qualitative analysis.

### Second step: co-occurrence networks

By design, the *contingent* lexicon escapes the SL, which cannot capture non-generic elements constituting the securitizing semantic repertoires. Furthermore, the SL does not reveal important parts of speech or stylistic devices, nor does it highlight the repertoire’s associative network or expose its semantic context. The second step of our method secures these three jobs simultaneously.

We use co-occurrence networks (semantic networks) to visualize a given repertoire’s most prominent terms and their relationships. Not yet used in securitization studies, this tool is a particularly powerful addition to ST’s methodological toolbox. Semantic networks, which are based on co-occurrence matrix tables, carry out two tasks simultaneously and allow for an intuitive visualization of both tasks’ results: they represent, in a single graph, the most frequent words of a corpus and show how often they are associated in sentences or paragraphs. Co-occurrence networks are therefore built to identify ‘policy frames’^54^ or political ‘narratives’ and ‘discourses’.^55^ They have recently been used to reveal dominant frames of health issues^56^ and to offer sharp comparative evidence of the differentiated framing of an epidemic outbreak (the 2015 measles epidemic) by internet users in two countries.^57^

In co-occurrence networks, each word is represented as a node whose size varies proportionally to frequency. The links between nodes represent their co-occurrence, with their thickness symbolizing the probability of the co-occurrence, and their length the average distance between the words. Co-occurrence networks therefore directly display the *associative networks* within a corpus, revealing a corpus’s most frequent terms in a way that allows for a qualitative analysis of *parts of speech* and *stylistic devices*. Clusters of frequently co-occurring words appear that approximate the presence of non-securitizing repertoires around the securitizing one(s), that is, the *semantic context* of the repertoire. To sum up, applying co-occurrence networks to securitizing speech displays not only the generic lexicon, common to most cases of securitization, but also the specific lexicon, particular to each case, as well as the meaning-giving relationships that connect the words of both lexicons.

For a given corpus, an option is to ‘seed’ a co-occurrence network, that is, to build a more focused network that displays the terms with the highest probability to be co-occurring with a certain word or set of words deemed theoretically important (the ‘seeded’ words). This technique allows a more granular visualization of the lexical field surrounding the seeded words, which is useful when one wants to zoom in on a particular object evoked in the corpus. Working on a corpus of ISIS propaganda magazines, researchers have for example generated a seeded network focusing on the word ‘West’ that allowed them to demonstrate how the organization frames the West as a decadent and aggressive ‘non-civilization’.^58^

For the present inquiry, we built two seeded networks – one for the US, one for the UK – visualizing the terms that have the highest probability to be co-occurring with the words ‘coronavirus’, ‘covid’ or ‘virus’ in the same paragraph.^59^ The networks include the top 150 edges between these words, a number found, after attempts with lower and higher numbers, to correspond to the ‘sweet spot’ between the amount of information provided and the readability of the graph.

### Corpus

To study the securitizing semantic repertoires of Covid-19, we compiled a structured corpus made of all recorded public statements mentioning the pandemic (‘virus’,^60^ ‘coronavirus’, ‘covid*’) made by US President Donald Trump and UK Prime Minister Boris Johnson. These two states were selected because, while neither initially opted for a clear securitization (or refusal thereof) of the disease,^61^ both changed their rhetoric at one point and implemented extraordinary measures. The rationale for selecting these otherwise different cases is thus to maximize the chance of detecting and visualizing these changes in the repertoires. The US and the UK also constitute seemingly anomalous cases: while the two countries were the best prepared to tackle a pandemic,^62^ with 535 and 610 deaths per million inhabitants respectively (as per 20 August 2020) they both rank in the top-10 of worst-hit countries in the world at the time of writing. While we neither examine here the policies taken or not by the Trump and Johnson administrations, nor establish a causal chain from securitizing language to these policies, our analysis nonetheless provides a first step towards understanding this puzzle.

Our corpus starts at the end of January 2020 for the US, and at the beginning of March 2020 for the UK, with a common end date matching Johnson’s speech declaring the first partial opening of the lockdown, and Trump’s announcement of the implementation of a large-scale testing strategy. This focus on the early months of the pandemic is warranted on two grounds: this timeframe not only incorporates the first speeches on the pandemic but also corresponds to the implementation of extraordinary measures in both countries. The data of the UK corpus included all official speeches from the Downing Street daily press briefings, as well as ad hoc official communications by the Prime Minister displayed on the government’s official website. The data of the US corpus consisted of the verbal interventions made by the US President during the White House press conferences, including his introductory speeches and answers to journalists, as well as official presidential speeches made on other occasions and available on the White House website. This data collection strategy resulted in a corpus of over 560,000 words, whose main statistics are provided in Table 2 above. Although Trump’s ‘tweets’ arguably constituted one of his most dominant forms of communication, we decided not to include them in the corpus for three main reasons. First, methodologically, we excluded them for the sake of comparability: a different media for a different purpose; Twitter entails a language that cannot be compared to that of official speeches. Second, theoretically, while leaders’ Tweets can arguably be considered as speech acts from the perspective of ST, their audience is never the entire nation but rather a community of followers, and their format implies a particular logic of message-reception; this is particularly true of the former US president, whose use of Twitter during the pandemic has been shown to be shaped by attention-seeking and persona-building considerations.^63^ Tweets therefore don’t involve the exact same dynamics of securitization than those of official speeches. Third, empirically, several studies already cover Donald Trump and other G7 leaders’ Tweets during the pandemic, showing for example that the US president’s tweets were much more likely to politicize the pandemic than those of his counterparts,^64^ or that his output became increasingly negative when it mentioned the coronavirus and China together.^65^ Our analysis complements this work by focusing on a type of messaging not yet investigated.

**Table 2.** Key information, US and UK corpora.

	Start–End	# Words	# Segments
US corpus	29/01/2020–11/05/2020	487.642	68
UK corpus	03/03/2020–11/05/2020	73.111	58

Keeping in mind that the two countries’ diverging communication strategies and traditions make the corpora not perfectly comparable, we took several decisions to allow, if not for a perfect, at least for a meaningful comparison. First, Boris Johnson’s illness and subsequent hospitalization mean that the UK corpus contains, from 26 March 2020, speeches made by key members of the restricted cabinet (e.g. Home Secretary, Chancellor, Health Secretary) without the Prime Minister being present himself. As we will see, this is partially reflected in the securitizing repertoire. Second, Covid speeches in the UK followed a standardized format that left little room for improvization, or for discussing other political issues, whereas the US president’s interventions lacked a recurring structure. This means that some of the US president’s interventions were very long and other extremely short, with longer speeches regularly occurring during questions time and frequently veering off-course on other topics. This results in the US section of the corpus being much longer than the UK one, as shown by Table 2. Because the SL is a ratio and not an absolute count, this discrepancy only marginally affected our findings. However, as we illustrate below, an extremely short speech containing just a few relevant words will inevitably score a very high ratio; this actually constitutes a theoretically important point related to the *semantic context* evoked above.

It should be noted that the following analysis limits its ambition to the official securitizing semantic repertoires of Trump and Johnson. As scholars such as Stritzel and Chang have rightly shown, official securitizing speech acts and practices are always parts of ‘prolonged political game[s] of moves and countermoves marked by securitizing and counter-securitizing speech acts’.^66^ Analyses of official communications during the previous pandemics indeed revealed how different political/social actors put forward differing discursive practices.^67^ Our aim here is not to open the lens to encompass what Chong and Druckman call the ‘competitive elite environment’ of framing,^68^ but rather to narrow the focal point to the central, most important repertoire defining the political game.

## Results: mapping and tracing the securitizing semantic repertoires of Covid-19

We discuss our results in two main sections corresponding to the computational tools described above, with the qualitative analysis of relevant segments directly embedded in the presentation. Overall, we make two main observations. First, the intensity of securitizing language has generally been low in both the UK and the US, yet with several spikes evidencing particularly important securitizing speech acts. Second, and even though the UK corpus displays a higher saliency of the generic security lexicon, the classical war metaphor is more prevalent in the US corpus. The UK, by contrast, has used a very specific contingent securitizing repertoire articulating a different logic than the one implied by the war metaphor: the biopolitical imperative to ‘save lives’. This has been done in a systematic and sustained way, whereas the use of the war metaphor in the US has been sporadic and diluted in a disordered semantic repertoire.

### Dictionary-based approach: measuring the intensity of securitizing language

**Figure 2.**
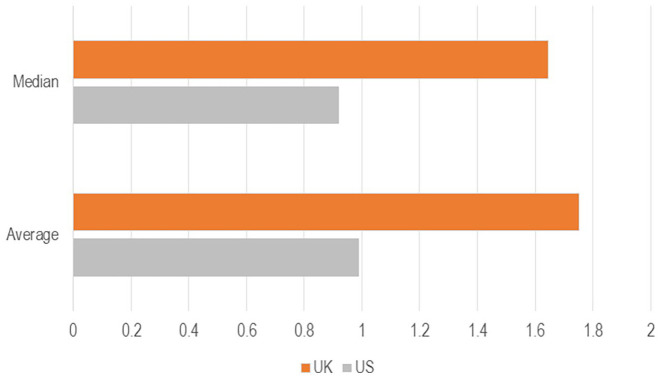
Descriptive statistics, SL in the US (grey) and the UK (orange) corpora.

**Figure 3.**
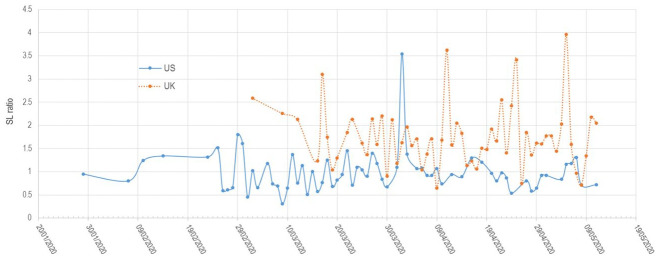
Salience of security lexicon across time, the US (blue, continuous) and the UK (orange, dashed) corpora.

Our dictionary-based analysis of the two corpora reveals three main results (see Figures 2 and 3 below). First, the intensity of the generic security lexicon (SL) has been much higher in the UK than in the US. The average saliency is almost twice as high (1.75 > 0.99), and so is the median score for each segment (1.64 > 0.92). This distinction maintains over time, with only rare incursions of the US line above the UK one. This difference echoes previous studies on the variable levels of securitization of epidemics found in different countries.^69^

Second, while these ratios undoubtedly denote the presence of securitizing language, they are surprisingly low in absolute terms. Indeed, previous uses of the SL show, on the one hand, that ‘fully securitized’ texts, such as EU directives directly dedicated to so-called ‘hard’ security matters, obtain scores around 5. On the other hand, a very large random corpus received a score of 1.26.^70^

These findings indicate that there has not been a strong and sustained securitization of the pandemic by the UK and US heads of government, at least through the use of the hard security words included in the generic lexicon. The absence of high and sustained levels of securitizing language, which would have reflected a stronger and less ambiguous stance on the virus, might constitute a small but non-negligible part of the answer to the puzzle of the two states’ poor performance in limiting fatalities.

Third, the analysis shows the presence of sharp ‘spikes’, that is, particular speeches that contain a higher-than-normal ratio of words from the generic security lexicon. Such an observation is important as it potentially reveals unique speeches that may correspond to the kind of clear securitizing speech acts initially envisioned by the Copenhagen School.

A qualitative examination of these specific speeches is therefore warranted. The US corpus has a generally low SL but displays a single spike at 3.54 – more than three times its average. This spike matches a statement given by Donald Trump on 2 April 2020, where he announced an executive order to bring production of medical supplies within the realm of the Defense Production Act. We transcribe the speech in extenso below:

Today, I have issued an order under the Defense Production Act to more fully ensure that domestic manufacturers can produce ventilators needed to save American lives. My order to the Secretary of Health and Human Services and the Secretary of Homeland Security will help domestic manufacturers like General Electric, Hill-Rom, Medtronic, ResMed, Royal Philips, and Vyaire Medical secure the supplies they need to build ventilators needed to defeat the virus. I am grateful to these and other domestic manufacturers for ramping up their production of ventilators during this difficult time. Today’s order will save lives by removing obstacles in the supply chain that threaten the rapid production of ventilators.

This speech has three major features that make it a clear securitizing move in the sense found in the original ST literature. First, Trump’s use of generic security words is high (‘defence’, ‘security’, ‘secure’, ‘defeat’, ‘threaten’ are all in the SL dictionary). Second, the speech is very short and straightforward, with no other issues discussed. In other words, the semantic context is empty, giving the impression of a sharp and important statement. Together, these first two features explain the high SL score (ratio of generic security words per total words). This is an interesting insight, which would require further investigation: the relative strength of a securitizing repertoire is higher when the repertoire is isolated than when a comparable repertoire co-exists alongside other repertoires in longer speeches. Third, this speech does in fact operationalize an extraordinary measure through executive action: the nationalization of economic production is a decision that stands out of normal politics, even more so in the US. In this regard, this is a typical example of a securitizing move in the original acceptation of the term by the Copenhagen School. Yet at the same time, attuning to the context and processes leading up to this speech helps us understand why it is at odds with the remainder of the corpus: while clearly securitizing, this statement was mostly made by Trump in reply to mounting pressures to do so, and was not followed by thorough measures based on the DPA (Trump did evoke the DPA and quickly signed an executive order to secure the production of ventilators, but did not use it extensively afterwards).^71^ So even though the speech uses securitizing language to acknowledge particular possibilities opened by the DPA, the speeches and actions that followed fell back to the initial line of very limited securitization.

Unlike the US corpus, the UK corpus has four spikes. The first spike corresponds to Boris Johnson’s pivotal speech on 17 March, which matches the implementation of lockdown. In this speech, Johnson makes a heavy use of the generic security lexicon, with words like ‘dangerous’, ‘overwhelm’, ‘war’ or ‘enemy’ pointing to a classic use of the war metaphor. ‘We must act like any wartime government’, Johnson proclaimed, with ‘a sense of urgency’. The historical analogy with World War II is used to allow the audience to instantly appraise the seriousness of the crisis, and the necessity of the extraordinary measures to come. Johnson uses many parts of speeches that do not appear in the generic dictionary but that further accentuate his securitizing move: adjectives such as ‘drastic’, ‘deadly’, ‘extreme’, ‘fast’, ‘severe’ or ‘unprecedented’; adverbs such as ‘quickly’; nouns like ‘fight’ or ‘crisis’; and verbs like ‘beat’ or ‘shielding’. All these parts of speech reinforce the sense of urgency and threat. Crucially, this speech corresponds, like Trump’s 2 April speech, to the arrival of what Johnson himself call ‘extreme measures’, which unambiguously belong to the realm of extraordinary politics. It therefore represents a clear case of a securitizing speech act as originally conceptualized by the Copenhagen School, and further demonstrates the usefulness of the SL to spot such language.

Yet the three other spikes in the UK corpus do not match with speeches of similar rhetoric. This is explained by the fact that, on 11 and 15 April 2020, Priti Patel delivered the speeches, in lieu of Johnson (see above): as Secretary of State for the Home Department, her addresses had a strong emphasis on Covid-related criminality. The 5 May 2020 speech, delivered by Secretary of State for Foreign and Commonwealth Affairs Dominic Raab, contained a series of statements related to UK-US collaboration on cyber-attacks that drove the SL higher. While making use of many generic security words, these are not securitizing speech acts.

In sum, the SL dictionary proved a powerful tool in three ways. First, it demonstrated that the intensity of generic securitizing language was surprisingly low. Second, it showed that the UK scores were significantly higher than those of the US. Third, it successfully identified a – perhaps *the* – key speech in each corpus, with the qualitative analysis showing that both speeches clearly match the sort of securitizing speech act originally conceptualized by the Copenhagen School and make use of what has been presented as the main metaphor of securitizing repertoires: war. However, as explained earlier, this approach is only an evaluation of the generic lexicon; it ignores the more contingent language participating to securitization. And it also brought about a puzzle: why is there such a difference in the SL ratio, with UK scores consistently higher than US scores? The second step of our analysis turns to the contingent lexicon and explains this puzzle.

### Co-occurrence networks: attuning to the contingent repertoire, identifying the referent object

**Figure 4.**
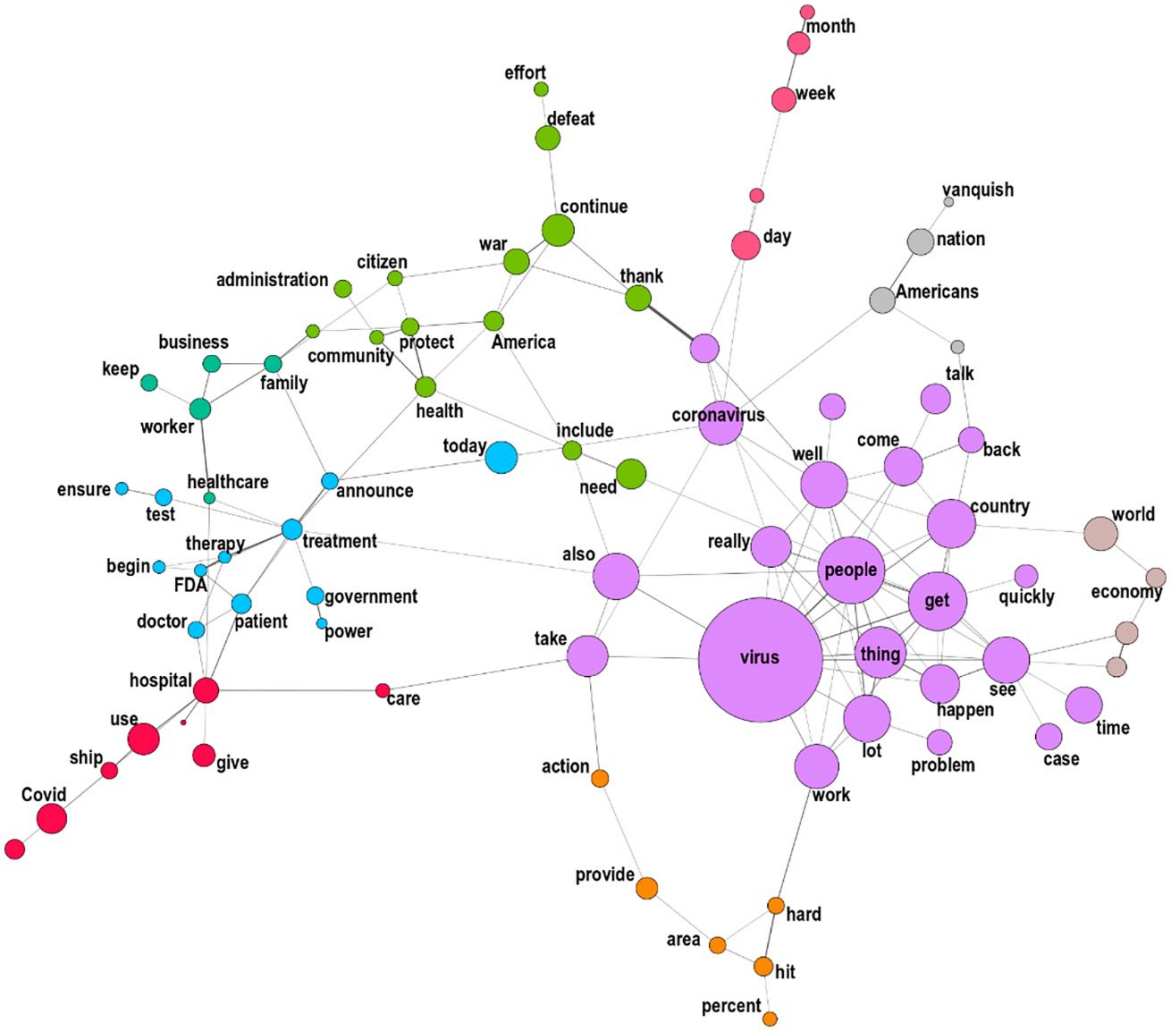
‘Virus’ co-occurrence network, US corpus.

Generating seeded co-occurrence networks allows for a granular analysis of exactly *how* the virus is portrayed in both countries. In other words, it allows us to detect and visualize the different repertoires used to securitize it. Figure 4 below represents the US network. Two main observations can be made. First, a cluster (green, top centre) centres around the war metaphor discussed above: this shows that the terms ‘war’, ‘defeat’, ‘protect’, ‘America’ and ‘citizens’ are frequently occurring together when the virus is discussed. This cluster relates to another, smaller, group of words sharing the same generic security lexicon (grey, top right: ‘vanquish’, ‘nation’, ‘Americans’). In fact, in the US corpus ‘defeat’ and ‘war’ are no less than the third and fourth terms with the highest probability to co-occur with ‘virus’, ‘coronavirus’ or ‘covid’. This shows the significance of the war metaphor as a key stylistic device used by Trump. As such, this illustrates a classical securitization move where the *referent object*, that is, what is being threatened and needs protection, is the state.

A qualitative reading of the texts containing the word ‘war’ shows that the US president used two historical analogies several times to add resonance to the war metaphor: Pearl Harbor and 9/11. These analogies lead the audience to read the Covid-19 crisis as a surprise attack on American soil that requires a rapid and uncompromising reaction. This use of the war metaphor as a structuring stylistic device parallels the militaristic tone of Israeli and Jordanian leaders highlighted by Hoffman, with the Israeli Defence Minister linking the ‘First Corona War’ to the ‘previous Israeli wars’ and the Jordanian King Abdullah II evoking, while wearing a military uniform, the 1968 War of Attrition and battle of Karameh.^72^ The US historical references are also made in reference to an enemy, which is blamed for the situation: China. This is consistent with Buzan, Waever and De Wilde’s intuition that ‘whatever is presented as the cause of security problems is most likely also actorized’.^73^ As it goes in the theory, the source of the threat is presented as an agent who made a choice that led to the untoward situation:

This is really the worst attack we’ve ever had. This is worse than Pearl Harbor. This is worse than the World Trade Center. There’s never been an attack like this. And it should have never happened. It could have been stopped at the source. It could have been stopped in China. It should have been stopped right at the source, and it wasn’t. [. . .] I view the invisible enemy as a war. I don’t like how it got here because it could have been stopped. But, no, I view the invisible enemy like a war. Hey, it’s killed more people than Pearl Harbor, and it’s killed more people than the World Trade Center. World Trade Center was close to 3,000. Well, we’re going to beat that by many times, unfortunately. So, yeah. This — we view it as a war. This is a mobilization against a war. It’s a — in many ways, it’s a tougher enemy. (6 May 2020)

Second, it is striking to note that the US securitizing repertoire is merely one among several others, less (or even non-) securitizing repertoires. Two clusters show the importance of a medico-scientific discourse centred chiefly on therapies and treatments (blue and pink, bottom left), but also economic considerations (green, left). In addition, a lot of un-thematic, some might even say ‘empty’, talks took place, which is evidenced by the biggest purple group of words on the right (among the most frequent terms and expressions in the US corpus are ‘things’, ‘lots of things’, ‘a lot of people’, ‘good job’, etc.). Such a large amount of unspecified language when talking about the virus, together with the coexistence of several non-securitizing repertoires, had, we argue, a major effect: it significantly diluted the securitizing language within a mass of talk which was either non-securitizing or ‘empty’.

In sum, the co-occurrence network analysis of the US corpus does reveal the presence of a securitizing semantic repertoire centred on the war metaphor and articulating a series of powerfully securitizing historical analogies. However, it shows simultaneously that this repertoire is diluted alongside other less (or non-) securitizing repertoires and a large amount of un-thematic talk.

**Figure 5.**
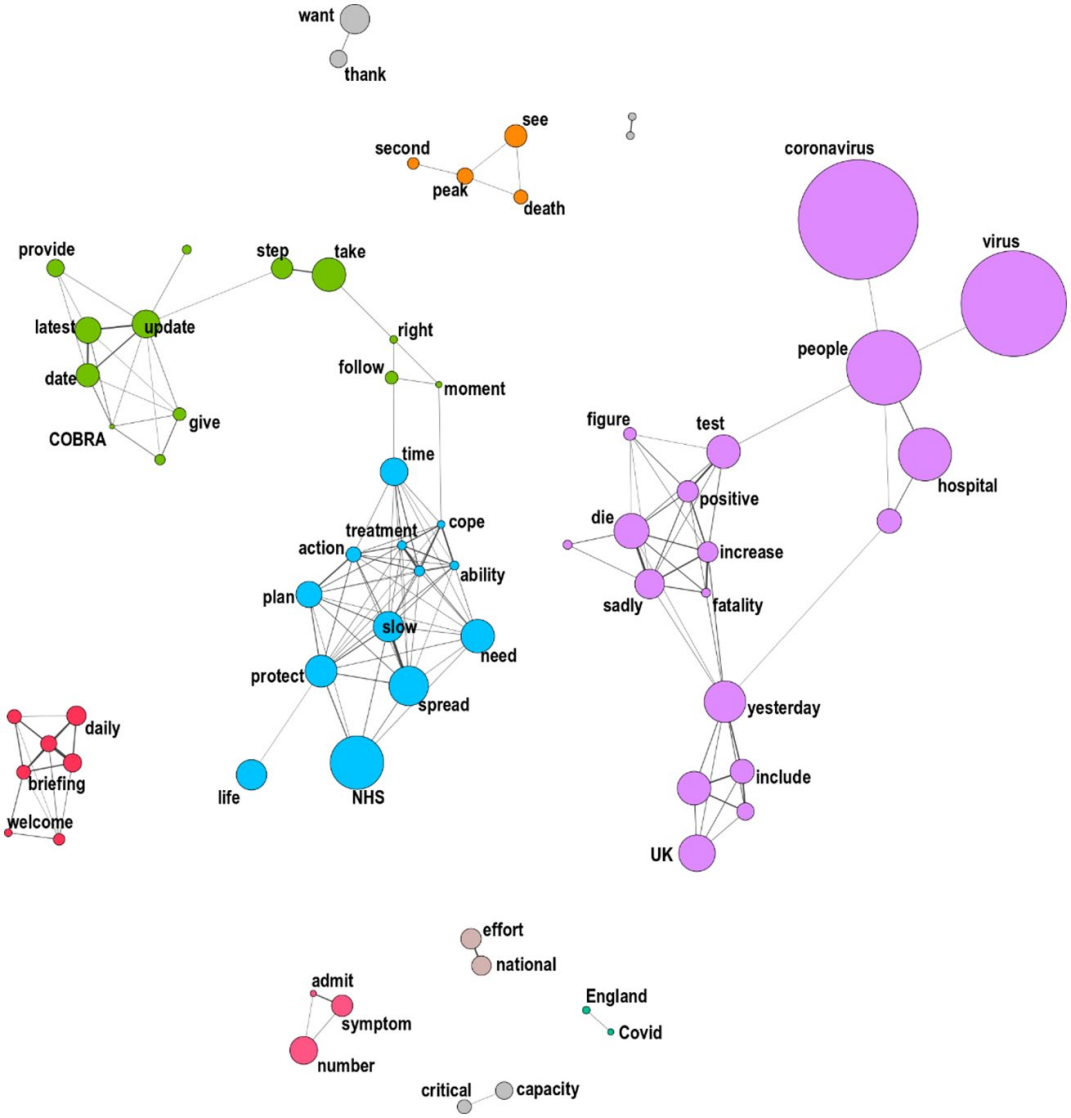
‘Virus’ co-occurrence network, UK corpus.

Turning to the UK corpus, Figure 5 below offers a very contrasting view. We notice that the war metaphor, even if used in Johnson’s pivotal 17 March speech, does not appear. That means that this speech was an outlier in terms of the type of securitizing semantic repertoire used (the term ‘war’ appears only 10 times in the corpus and ‘wartime’ just once; ‘threat’ occurs only 12 times and ‘security’ 10 times). This also means, given the high SL scores, that another securitizing lexicon must be present and at least partially overlapping with a specific generic lexicon that does not articulate the classic war metaphor.

The network clearly shows the coherence of the semantic repertoire used by the UK leaders: in contrast to the US network, all clusters link scientific-medical terms, with no other issue raised when discussing the virus, and little empty talk. UK officials focus on the pandemic figures (testing, fatalities, etc.) and people admitted to hospitals (purple, right), the government’s action plan to slow the spread of the epidemic and thereby reduce the strain on the NHS (blue, central) and the efforts to prevent deaths from a second peak (orange, top). This is where the specificity of the UK’s securitizing repertoire lies: rather than resting on a classic, hard security lexicon centred on the war metaphor and related stylistic figures and parts of speech, it uses a range of securitizing terms related to *life and death*. The repertoire therefore comes closer to Huysmans’s understanding of security as ‘a strategy constituting and mediating our relation to death’.^74^ Words like ‘die’, ‘fatality’ or ‘life’ are among the most likely to co-occur in a paragraph discussing the virus, and have an obvious securitizing potential. While some of these terms, chiefly ‘protect’, actually belong to the generic dictionary, and explain the consistently high UK score, most do not, and thus constitute a contingent securitizing semantic repertoire, which articulates a biopolitical logic.^75^ This logic rests on the diffusion and internalization of norms of self-care and well-being as well as on the sacralization of life, rather than on hard security measures necessarily involving heavy sacrifices and life loss. Biopower, as Foucault summarized, is a ‘technology of power which takes life as both its object and its objective, [. . .] its basic function is to improve life, to prolong its duration, to improve its chances, to avoid accidents, and to compensate for failings’.^76^

Echoing Elbe’s assimilation of the securitization of HIV/AIDS to biopower,^77^ we observe here that, unlike the US, the UK has centred its speech about the virus on the biopolitical necessity to ‘save lives’. This tendency, which became fully dominant after the UK government read a model predicting 260,000 deaths,^78^ is encapsulated in the last tenet of the constantly repeated and ‘too successful’^79^ triad ‘Stay Home, Protect the NHS, Save Lives’. To translate this in the terms of ST, the *referent object* of the UK securitizing move on Covid-19 is not the state – like with the war metaphor – but the individual and the institution that protects his or her life, the NHS.

The UK securitizing repertoire constitutes the epitome of what Rose coined ‘risk politics’, that is, a particular accentuation of biopolitics that has developed in advanced liberal societies, whereby political strategies aimed at maximizing life rest on ‘calculations about probable futures in the present, followed by interventions into the present in order to control that potential future’.^80^ With biopolitical risk, policy decisions differ from those taken by governments that adopt a more disciplinary stance, as the aim shifts from ‘waging war from the defence of the sovereign to securing the existence of a population’.^81^ The onus is on citizens to ‘become an active partner in the drive for health, accepting their responsibility for securing their own well-being, [. . .] the health-related aspirations and conduct of individuals is governed “at a distance”, by shaping the ways they understand and enact their own freedom’.^82^ Predictive surveillance and monitoring systems to which citizens themselves subscribe and share their data are the typical policy of such a logic of government. This UK stance is not unprecedented. Indeed, the biopolitical approach to pandemics has already been noticed in the cases of H1N1^83^ and HIV/AIDS.^84^

This is where the semantic difference between the US and UK repertoires really matters. On the one hand, Trump developed a hard security frame constructing the pandemic as a warlike attack justifying a retaliatory action against an outside enemy, a top-down hierarchy, and disciplinary measures, with more consideration to sacrifice in order to save the US referent object, the state, than to the preservation of life. This stands in line with the US president’s usual emphasis on law and order, and hard borders, but also with a pre-election context pushing him to deflect the blame on others, chiefly China. On the other hand, the UK government adopted a biopolitical framing urging individuals to participate in the maximization of life.

Both repertoires are securitizing, and have been used to move the pandemic out of the realm of ordinary politics, but their underpinning logic, consequences and referent objects are different. Of course, as Knauft warned the two logics are never purely at play in contemporary societies, but rather always enmeshed into new power-knowledge nexuses.^85^ Johnson used the war metaphor in what was arguably his strongest speech, while Trump did discuss fatality rates. It remains, though, that our analysis reveals how both states relied on noticeably different securitizing semantic repertoires.

## Conclusions

In this paper, we strengthened Securitization Theory’s conceptualization of its pivotal component, securitizing language, by putting forward a consolidated theory of securitizing semantic repertoires. We suggested a multi-dimensional model and used this framework to study the securitization of Covid-19 in the US and the UK during the initial period of the pandemic: from January (for the US) and March 2020 (for the UK) until May 2020. This focus allows us to shed light on the controversy centred on whether the two governments, who ranked as first and second states the best prepared to deal with a major outbreak, had initially downplayed the risk of the disease. Accordingly, we did not aim to offer an overview of the whole securitization process, but rather to clarify as much as possible the pivotal moment when a securitizing actor (the head of state) frames an object (Covid-19) as an existential threat requiring the implementation of extraordinary measures in order to protect a referent object. Thanks to a combination of dictionary-based and co-occurrence analyses reinforced by qualitative checks – a combination which, we believe, has the potential to solve some of the methodological shortcomings of securitization research – our multidimensional theory allowed us to evaluate both the *intensity* and *way* in which Donald Trump and Boris Johnson have securitized the virus in their public speeches.

With regard to the *intensity*, we found, first, that the UK corpus displayed a higher saliency of the generic lexicon compared to the US, and that this tendency was consistent over time. Second, and despite this observation, we showed that neither leader has opted in favour of a strong and sustained securitization of the disease, the ratio of their securitizing language remaining relatively low. Despite this low intensity, our analysis allowed us to spot several spikes evidencing particularly significant securitizing speech acts. Third, we conducted a qualitative analysis to investigate these particular speeches and found out that they tended to correspond to the arrival of extreme measures to deal with the threat; thereby showing the effectiveness of our model in identifying these key moments. Our US data also indicated that the relative strength of a securitizing repertoire is higher when the repertoire is isolated, like with Trump’s 2 April statement, compared to when the same repertoire co-exists alongside other repertoires in longer speeches.

Our seeded co-occurrence analysis shows significant variation in the *way* both leaders have securitized Covid-19 in their public speeches. In particular, we found out that the securitizing repertoire mobilized by each leader was framed around the protection of a different referent object. On the US side, we first revealed the presence of a securitizing semantic repertoire making use of the war metaphor and articulating a series of powerfully securitizing historical analogies, as well as the identification of China as the bearer of blame for the situation. This latter finding, in particular in a pre-election context, suggests an interesting link between securitization and blame avoidance behaviours that ought to be further investigated. Second, while the presence of this ‘hard security’ repertoire was undeniable in the US, it was diluted alongside other less (or non-) securitizing repertoires, and a large amount of un-thematic talk, which links back to, and explains, the low intensity of the US securitizing language. On the UK side, we identified what could be called a ‘biopolitical repertoire’, centred on the preservation of life. Accordingly, to go back to ST, while the referent object in the US repertoire seems to be the state under attack, the referent object in the UK repertoire is the individual and the institution that protects its life, the NHS. Finally, unlike the US corpus, this repertoire is not scattered and diluted but exists in an orderly and structured manner in the speeches of Johnson and UK cabinet members.

Altogether, these findings lead us to the compelling observation that other repertoires than those centred on ‘hard security’ war metaphor can be used to securitize an issue. In our comparison, the biopolitical repertoire of the UK even scores higher on a generic measurement of securitizing language than the US war repertoire.

In spite of these clear findings, our intervention does not close the debate on the language of securitization or the Covid-19 case. *Theoretically speaking*, a case like the Covid-19 pandemic seems to bring back with force the initial formulation of ST by the Copenhagen School, with its emphasis on well-delineated speech acts followed by extraordinary measures. This does not mean, however, that other approaches to securitization are wrong, but rather than ST is best understood as a plural framework^86^ containing several lenses, each one of them best suited to explain and interpret a specific case. *Empirically speaking*, further research should widen the empirical universe covered here. Other cases, for example including countries that notoriously resisted the language of war and security, like Sweden or New Zealand, would need to be examined in order to fine-tune the theory of securitizing repertoires. An analysis of the ‘visual repertoire’ of Covid-19 could also be carried out, investigating how pictures of biosuits, extenuated doctors and nurses, and other prominent visual tropes connect with securitizing language.^87^ An evaluation of the actual impact of various securitizing repertoires on different audiences, as well as the tracing of the dissemination and modifications of these repertoires when echoed by the press or social media, could be undertaken. Relatedly, and as acknowledged, the question of the relationship between securitizing repertoires and the policies adopted is voluntarily left unexplored here, as we primarily sought to strengthen the theory’s take on language, but could become the focus of future research.

## Notes

1. Barry Buzan, Ole Wæver and Jaap de Wilde, *Security: A New Framework for Analysis* (Boulder, CO: Lynne Rienner, 1998), p. 36.

2. Buzan et al., *Security*, p. 24.

3. Our translation. For the full speech, see Elysée, ‘Coronavirus Covid 19’, available at: https://www.elysee.fr/emmanuel-macron/coronavirus-covid-19 (accessed 1 June 2020).

4. Both examples (and translations) are from Adam Hoffman, ‘The Securitization of the Coronavirus Crisis in the Middle East’, *POMEPS Studies*, 39 (The COVID-19 Pandemic in the Middle East and North Africa), 2020, pp. 10–4.

5. Blog contributions have already examined the securitization of the coronavirus, for example Saud Al Sharafat, ‘Securitization of the Coronavirus Crisis in Jordan: Successes and Limitations’, Washington Institute, 11 May 2020, available at: https://www.washingtoninstitute.org/fikraforum/view/COVID-19-Jordan-Middle-East-Securitization (accessed 1 June 2020); Nathan Sears, ‘The Securitization of COVID-19: Three Political Dilemmas’, Global Policy, 25 March 2020, available at: https://www.globalpolicyjournal.com/blog/25/03/2020/securitization-covid-19-three-political-dilemmas (accessed 1 June 2020).

6. Buzan et al., *Security*, pp. 24–5.

7. Read for example Catarina Thomson and Stephane Baele, ‘Securitization Theory’, in Allan Collins (ed.) *Contemporary Security Studies*, 6th ed. (Oxford: Oxford University Press, 2022), pp. 173–85.

8. Stephane Baele and Diana Jalea, ‘Twenty-Five Years of Securitization Theory: A Corpus-Based Review’, *Political Studies Review*. Epub ahead of print 20 January 2022. DOI: 10.1177/14789299211069499.

9. The corpus containing these speeches is available upon request to the authors.

10. Such as Stefan Elbe, ‘AIDS, Security, Biopolitics’, *International Relations*, 19(4), 2005, pp. 403–19; Stefan Elbe, ‘Should HIV/AIDS Be Securitized? The Ethical Dilemmas of Linking HIV/AIDS and Security’, *International Studies Quarterly*, 50(1), 2006, pp. 119–44; Colin McInnes and Simon Rushton, ‘HIV/AIDS and Securitization Theory’, *European Journal of International Relations*, 19(1), 2013, pp. 115–38; or Melissa Curley and Jonathan Herington, ‘The Securitization of Avian Influenza: International Discourses and Domestic Politics in Asia’, *Review of International Studies*, 37(1), 2011, pp. 141–66.

11. Ole Wæver, ‘Securitization and Desecuritization’, in Ronnie D. Lipschutz (ed.), *On Security* (New York, NY: Columbia University Press, 1995), pp. 46–86; Buzan et al., *Security*. Sociological variants of ST emphasize the role of non-linguistic practices in securitization processes; for a discussion see Thierry Balzacq, ‘The “Essence” of Securitization: Theory, Ideal Type, and a Sociological Science of Security’, *International Relations*, 29(1), 2015, pp. 103–13. While the present effort aims at strengthening the language-based approach to ST, its framework and findings are useful for other takes on the theory.

12. Thierry Balzacq, ‘A Theory of Securitization: Origins, Core Assumptions, and Variants’, in Thierry Balzacq (ed.), *Securitization Theory: How Security Problems Emerge and Dissolve* (London: Routledge, 2011), p. 3.

13. Michael Williams, ‘Words, Images, Enemies: Securitization in International Politics’, *International Studies Quarterly*, 47(4), 2003, p. 513.

14. Jef Huysmans, ‘What’s in an Act? On Security Speech Acts and Little Security Nothings’, *Security Dialogue*, 42(4–5), 2011, p. 371.

15. For example Holger Stritzel, ‘Securitization, Power, Intertextuality: Discourse Theory and the Translations of Organized Crime’, *Security Dialogue*, 43(6), 2012, pp. 549–67; Julie Wilhelmsen, ‘How Does War Become a Legitimate Undertaking? Re-engaging the Post-Structuralist Foundation of Securitization Theory’, *Cooperation & Conflict*, 52(2), 2017, pp. 166–83.

16. Wæver, ‘Securitization and Desecuritization’, p. 55.

17. Buzan et al., *Security*, pp. 24–5.

18. Buzan et al., *Security*, p. 27.

19. See, for example, Mike S. Schäfer, Jürgen Scheffran and Logan Penniket, ‘Securitization of Media Reporting on Climate Change? A Cross-National Analysis in Nine Countries’, *Security Dialogue*, 47(1), 2016, 76–96.

20. Thierry Balzacq, ‘The Three Faces of Securitization: Political Agency, Audience and Context’, *European Journal of International Relations*, 11(2), 2005, p. 179.

21. Balzacq, ‘The Three Faces of Securitization’, p. 180.

22. Balzacq, ‘A Theory of Securitization’, p. 11.

23. Fred Vultee, ‘Securitization as a Media Frame’, in Thierry Balzacq (ed.), *Securitization Theory: How Security Problems Emerge and Dissolve* (London: Routledge, 2011), pp. 77–93.

24. Philipp Klüfers, ‘Security Repertoires: Towards a Sociopragmatist Framing of Securitization Processes’, *Critical Studies on Security*, 2(3), 2014, pp. 278, 282.

25. Stephane Baele and Olivier Sterck, ‘Diagnosing the Securitisation of Immigration at the EU Level: A New Method for Stronger Empirical Claims’, *Political Studies*, 63, 2015, p. 1124.

26. The pivotal moment of the securitizing speech act is not solely linguistic. Other dimensions, such as the speaker’s display of emotions for example, or the decorum surrounding the speech, also require more in-depth investigations that are beyond the scope of the present paper.

27. For a review, see Andreas Musolff, ‘Metaphor and Persuasion in Politics’, in Elena Semino and Zsófia Demjén (eds), *Routledge Handbook of Metaphor and Language* (New York, NY: Routledge, 2016), pp. 327–40.

28. George Lakoff, ‘The Contemporary Theory of Metaphor’, in Andrew Ortony (ed.), *Metaphor and Thought* (Cambridge: Cambridge University Press, 1993), p. 203.

29. Emily Crick, ‘Drugs as an Existential Threat: An Analysis of the International Securitization of Drugs’, *International Journal of Drug Policy*, 23(5), 2012, pp. 407–14; Heinz Steinert, ‘The Indispensable Metaphor of War: On Populist Politics and the Contradictions of the State’s Monopoly of Force’, *Theoretical Criminology*, 7(3), 2003, pp. 265–91.

30. Stephen Flusberg, Teenie Matlock and Paul Thibodeau, ‘War Metaphors in Public Discourse’, *Metaphor & Symbol*, 33(1), 2018, pp. 1–18.

31. Flusberg et al., ‘War Metaphors’, p. 6.

32. Flusberg et al., ‘War Metaphors’, pp. 4–6.

33. For example, Djouaria Ghilani, Olivier Luminet, Hans-Peter Erb, et al., ‘Looking Forward to the Past: An Interdisciplinary Discussion on the Use of Historical Analogies and Their Effects’, *Memory Studies*, 10(3), 2017, pp. 274–85.

34. Yuen Foong Khong, *Analogies at War* (Princeton, NJ: Princeton University Press, 1992).

35. For example, Robin Engström, ‘The Body Politic of Independent Scotland: National Personification and Metaphor as Ideological Visions’, *Metaphor & Social World*, 8(2), 2018, pp. 184–206.

36. James Pennebaker, Matthias Mehl and Kate Niederhoffer, ‘Psychological Aspects of Natural Language Use: Our Words, Our Selves’, *Annual Review of Psychology*, 54, 2003, pp. 547–77.

37. James Pennebaker, *The Secret Life of Pronouns: What Our Words Say About Us* (London: Bloomsbury, 2013).

38. For example, Ziva Kunda, *Social Cognition: Making Sense of People* (Cambridge, MA: MIT Press, 1999), pp. 42–51.

39. Aimei Yang and Charles Self, ‘Anti-Muslim Prejudice in the Virtual Space: A Case Study of Blog Network Structure and Message Features of the “Ground Zero Mosque Controversy”’, *Media, War & Conflict*, 8(1), 2015, pp. 46–69.

40. This suggestion echoes Hansen’s proposal to study the “processes of linking” in security texts; see Lene Hansen, *Security as Practice: Discourse Analysis and the Bosnian War* (New York, NY: Routledge, 2006), p. 17.

41. Although the media’s role of reformulating or highlighting securitizing speech-acts can be important, see Vultee, ‘Securitization as a Media Frame’.

42. Thierry Balzacq, ‘The Significance of Triangulation to Critical Security Studies’, *Critical Studies on Security*, 2(3), 2014, p. 378.

43. For example, Stephane Baele and Catarina Thomson, ‘An Experimental Agenda for Securitization Theory’, *International Studies Review*, 19(4), 2017, pp. 646–66. See also Georgios Karyotis and Stratos Patrikios, ‘Religion, Securitization and Anti-Immigration Attitudes: The Case of Greece’, *Journal of Peace Research*, 47(1), 2010, pp. 43–57.

44. For example, Paul Baker, Costas Gabrielatos, Majid Khosravinik, et al., ‘A Useful Methodological Synergy? Combining Critical Discourse Analysis and Corpus Linguistics to Examine Discourses of Refugees and Asylum Seekers in the UK Press’, *Discourse & Society*, 19(3), 2008, pp. 273–306. We also follow Lucas et al.’s demonstration that computer-assisted text analysis holds great potential for comparative political analysis: Christopher Lucas, Richard Nielsen, Margaret Roberts, et al., ‘Computer-Assisted Text Analysis for Comparative Politics’, *Political Analysis*, 23(2), 2015, pp. 254–77.

45. For example, Karyotis and Patrikios, ‘Religion, Securitization and Anti-Immigration Attitudes’; Ian Paterson and Georgios Karyotis, ‘“We Are, By Nature, A Tolerant People”: Securitisation and Counter-Securitisation in UK Migration Politics’, *International Relations*, 36(1), 104–26; Philippe Bourbeau, *The Securitization of Migration: A Study of Movement and Order* (New York, NY: Routledge, 2011).

46. Baele and Jalea, ‘Twenty-Five Years of Securitization Theory’, p. 11.

47. Leah Windsor, Nia Dowell, Alistair Windsor, et al., ‘Leader Language and Political Survival Strategies’, *International Interactions*, 44(2), 2018, pp. 321–36.

48. For example, Amir Razavi, Diana Inkpen, Sasha Uritsky, et al., ‘Offensive Language Detection Using Multi-level Classification’, in Atefeh Farzindar and Vlado Kešelj (eds), *Advances in Artificial Intelligence. Canadian AI 2010. Lecture Notes in Computer Science* (Heidelberg: Springer, 2010), pp. 16–27; Stéphan Tulkens, Lisa Hilte, Elise Lodewyckx, et al., ‘A Dictionary-Based Approach to Racism Detection in Dutch Social Media’, *arXiv* 1608.08738, 2016.

49. For example, Shuki Cohen, Arie Kruglanski, Michele Gelfand, et al., ‘Al-Qaeda’s Propaganda Decoded: A Psycholinguistic System for Detecting Variations in Terrorism Ideology’, *Terrorism & Political Violence*, 30(1), 2018, pp. 142–71.

50. Baele and Sterck, ‘Diagnosing the Securitisation’, pp. 1124, 1128.

51. James Pennebaker, Roger Booth, Ryan Boyd, et al., *Linguistic Inquiry and Word Count: LIWC2015. Operator Manual*, 2015, available at: https://www.liwc.app/static/documents/LIWC2015%20Manual%20-%20Operation.pdf (accessed 8 September 2022).

52. We removed the terms ‘biological’, ‘chemical’, and ‘drugs’ from the original dictionary. This was done for two reasons: first, these terms have no evident securitizing weight in the Covid-19 context; second, their high frequency in the corpus would have distorted the results.

53. Benjamin Smith, Michael Stohl and Musa al-Gharbi, ‘Discourses on Countering Violent Extremism: The Strategic Interplay Between Fear and Security After 9/11’, *Critical Studies on Terrorism*, 12(1), 2019, pp. 151–68.

54. Junseop Shim, Chisung Park and Mark Wilding, ‘Identifying Policy Frames Through Semantic Network Analysis: An Examination of Nuclear Energy Policy Across Six Countries’, *Policy Sciences*, 48, 2015, pp. 51–83.

55. Leo Kim, ‘Denotation and Connotation in Public Representation: Semantic Network Analysis of Hwang Supporters’ Internet Dialogues’, *Public Understanding of Science*, 22(3), 2013, pp. 335–50.

56. Leo Kim, ‘Media Framing of Stem Cell Research: A Cross-National Analysis of Political Representation of Science Between the UK and South Korea’, *Journal of Science Communication*, 10(3), 2011, pp. 1–15.

57. Lu Tang, Bije Bie and Degui Zhi, ‘Tweeting About Measles During Stages of an Outbreak: A Semantic Network Approach to the Framing of an Emerging Infectious Disease’, *American Journal of Infection Control*, 46(12), 2018, pp. 1375–80.

58. Stephane Baele, Gregorio Bettiza, Katharine Boyd, et al., ‘ISIS’s Clash of Civilizations: Constructing the “West” in Terrorist Propaganda’, *Studies in Conflict & Terrorism*, 44(11), 2021, pp. 887–919.

59. To harmonize and clarify the analysis we conflated the terms ‘covid-19’ and ‘covid’ in the corpus, while keeping ‘coronavirus’ and ‘virus’ distinct because of the different connotations they may possess (e.g., ‘Chinese virus’). We did not include the term ‘pandemic’, which occurs very few times; including it or not therefore does not meaningfully alter the network. The probability calculations were made using the Jaccard coefficient (full table available upon demand to the authors; for the calculations underpinning the probability table and the network, see Koishi Higushi, *KH Coder 3 Reference Manual*, 2017, available at: https://github.com/ko-ichi-h/khcoder (accessed 1 June 2020) as both tasks were carried out in the KHCoder 3.0). General co-occurrence networks for the entire corpora generated a range of issues that would have hampered their readability in the context of the current analysis.

60. We made sure that the virus mentioned was indeed Covid-19.

61. For instance, Trump claimed several times in January and February that the virus would be gone by April as temperatures would rise (see France24, ‘From “Hoax” to Pandemic: Trump’s Shifting Rhetoric on Coronavirus’, 20 March 2020, available at: https://www.youtube.com/watch?v=YWupoHcoLT4 (accessed 1 June 2020)), while, early March, Johnson still joked that he shook hands with everyone at a hospital ward and dismissed increased contaminations as ‘speculative’ (see Reuters, “Johnson listened to his scientists about coronavirus – but they were slow to sound the alarm”, 7 April 2020, available at: https://www.reuters.com/article/us-health-coronavirus-britain-path-speci/special-report-johnson-listened-to-his-scientists-about-coronavirus-but-they-were-slow-to-sound-the-alarm-idUSKBN21P1VF (accessed 1 June 2020)).

62. See for example Gavin Yamey and Clare Wenham, ‘The U.S. and U.K. Were the Two Best Prepared Nations to Tackle a Pandemic – What Went Wrong?’, 1 July 2020, available at: https://time.com/5861697/us-uk-failed-coronavirus-response/ (accessed 2 July 2020), based on the Global Health Security Index, available at: https://www.ghsindex.org/.

63. David Altheide, ‘Pandemic in the Time of Trump: Digital Media Logic and Deadly Politics’, *Symbolic Interaction*, 43(3), 2020, pp. 514–40.

64. For example, Sohaib Rufai and Catey Bunce, ‘World Leaders’ Usage of Twitter in Response to the COVID-19 Pandemic: A Content Analysis’, *Journal of Public Health*, 42(3), 2020, pp. 510–16.

65. Ussama Yaqub, ‘Tweeting During the Covid-19 Pandemic’, *Digital Government*, 2(1), 2021, pp. 1–7.

66. Holger Stritzel and Sean Chang, ‘Securitization and Counter-Securitization in Afghanistan’, *Security Dialogue*, 46(6), 2015, p. 548.

67. For example, on the H1N1: Charles Briggs and Mark Nichter, ‘Biocommunicability and the Biopolitics of Pandemic Threats’, *Medical Anthropology*, 28(3), 2009, pp. 189–98.

68. Dennis Chong and James Druckman, ‘A Theory of Framing and Opinion Formation in Competitive Elite Environments’, *Journal of Communication*, 57(1), 2007, pp. 99–118.

69. See Curley and Herington ‘The Securitization of Avian Influenza’.

70. For these scores, see Baele and Sterck, ‘Diagnosing the Securitisation’, and Smith et al., ‘Discourses on Countering Violent Extremism’.

71. See Altheide, ‘Pandemic’, p. 532; Aishvarya Kavi, ‘Virus Surge Brings Calls for Trump to Invoke Defense Production Act’, 22 July 2020, available at: https://www.nytimes.com/2020/07/22/us/politics/coronavirus-defense-production-act.html (1 August 2020).

72. Hoffman, ‘The Securitization of the Coronavirus Crisis in the Middle East’.

73. Buzan et al., *Security*, p. 44.

74. Jef Huysmans, ‘Security! What Do You Mean? From Concept to Thick Signifier’, *European Journal of International Relations*, 4(2), 1998, p. 233.

75. The concepts of biopolitics and biopower were developed by Foucault to depict the emergence of a logic of government geared at the maximization of a population’s health in liberal societies. See e.g., Michel Foucault, *Security, Territory, Population. Lectures at the College de France, 1977-1978* (London: Palgrave Macmillan, 2013).

76. Michel Foucault, ‘Society Must Be Defended’. Lectures at the College de France, 1975-76 (New York, NY: Picador, 2003), p. 254.

77. Elbe, ‘AIDS, Security, Biopolitics’.

78. Elisabeth Mahase, ‘Covid-19: UK Starts Social Distancing After New Model Points to 260 000 Potential Deaths’, *BMJ*, 368, 2020, p. m1089.

79. On the origins of this motto, see Christopher Hope and Hayley Dixon, ‘The Story Behind “Stay Home, Protect the NHS, Save Lives” – The Slogan That was Too Successful’, *The Telegraph*, 1 May 2020, available at: https://www.telegraph.co.uk/politics/2020/05/01/story-behind-stay-home-protect-nhs-save-lives/ (accessed 1 June 2020).

80. Nikolas Rose, ‘The Politics of Life Itself’, *Theory, Culture & Society*, 18(6), 2001, pp. 6–7; Ian Hacking, *The Taming of Chance* (Cambridge: Cambridge University Press, 1990).

81. David Campbell, ‘The Biopolitics of Security: Oil, Empire, and the Sports Utility Vehicle’, in Elizabeth Dauphinee and Cristina Masters (eds), *The Logics of Biopower and the War on Terror* (New York, NY: Palgrave MacMillan, 2007), pp. 129–56.

82. Rose, ‘The Politics of Life Itself’.

83. Briggs and Nichter, ‘Biocommunicability’.

84. Jaakko Ailio, ‘Liberal Thanatopolitics and the HIV/AIDS Pandemic’, *Alternatives*, 38(3), 2013, pp. 256–67.

85. Bruce Knauft, ‘On the Political Genealogy of Trump After Foucault’, *Genealogy*, 2(4), 2018, pp. 1–18.

86. Or, as Balzacq puts it, an ideal-type (Balzacq, ‘The “Essence” of Securitization’).

87. But also numerical data and representations such as charts and figures, or schematic representations of the virus.

